# Exergy valorization of a water electrolyzer and CO_2_ hydrogenation tandem system for hydrogen and methane production

**DOI:** 10.1038/s41598-019-42814-6

**Published:** 2019-04-23

**Authors:** Omar S. Mendoza-Hernandez, Asuka Shima, Hiroshige Matsumoto, Mitsuhiro Inoue, Takayuki Abe, Yoshio Matsuzaki, Yoshitsugu Sone

**Affiliations:** 1 Japan Aerospace Exploration Agency, 3-1-1 Yoshinodai, Chou-ku, Sagamihara, Kanagawa 252-5210 Japan; 2 International Institute for Carbon-Neutral Energy Research (WPI-I2CNER), Kyushu University, 744 Motooka, Nishi-ku, Fukuoka City, Fukuoka 819-0395 Japan; 3 Hydrogen Isotope Research Center, University of Toyama, Gofuku 3190, Toyama, 930-8555 Japan; 4 Fundamental Technology Department, Tokyo Gas. Co., Ltd., 1-7-7 Suehiro-cho, Tsurumi-ku, Yokohama City, Kanagawa 230-0045 Japan; 5 The Graduate University of Advanced Studies, SOKENDAI, 3-1-1 Yoshinodai, Chou-ku, Sagamihara, Kanagawa 252-5210 Japan

**Keywords:** Chemical engineering, Renewable energy

## Abstract

In this work, we introduce a water electrolysis and CO_2_ hydrogenation tandem system which focuses on methane generation. The concept consists of a water electrolyzer thermally coupled to a CO_2_ hydrogenation reactor, where the power required to generate hydrogen comes from renewable energy. A thermodynamic analysis of the tandem system was carried out. Our analysis exposes that it is possible to increase the exergy efficiency of the water electrolyzer and CO_2_ hydrogenation system by thermal coupling, where the thermal energy required to split water into H_2_ and O_2_ during the electrolysis process is compensated by the heat generated during the CO_2_ hydrogenation reaction. Here, the conditions at which high exergy efficiency can be achieved were identified.

## Introduction

According to measurements done by the greenhouse gases observing satellite (GOSAT), the concentration of CH_4_ and CO_2_ in earth’s atmosphere has been increasing continuously in the recent years^1^. This indicates that the exploitation of fossil fuels to generate the energy required to meet our modern needs is not only threating a shortage in our limited fuel sources but also damaging our environment. In order to decarbonize our energy supply, alternative energy technologies must be considered^2,3^. The use of hydrogen as energy carrier is a promising way to encourage the decarbonization of energy, since it can be produced from the splitting process of H_2_O (water electrolysis), which generates H_2_ and O_2_ without exhausting carbon to the environment as long as the energy required to achieve this process comes from renewable sources^4,5^. The hydrogen obtained from the electrolysis process can be used to hydrogenate a CO_2_ source where CH_4_ and H_2_O are produced, this process is known as Power-to-Gas (PtG)^6–10^. PtG is a technology that connects the electrical power grid with the gas lines by converting excess electricity into a gas that can be injected to the existing gas distribution pipelines.

The PtG process is similar to life support systems for closed environments like the one that is currently used at the International Space Station (ISS), where O_2_ and H_2_O are the desired products to support life^11,12^. O_2_ and H_2_ are obtained from water electrolysis. H_2_ is used to convert the metabolic CO_2_ generated by the ISS crew to produce H_2_O which generates CH_4_ as a byproduct. The obtained H_2_O can be used to generate O_2_ while the obtained CH_4_ is vented to outer space.

Power-to-Gas technology is a promising alternative to recycle a global CO_2_ and reduce carbon emissions to our atmosphere, especially when the energy required to generate H_2_ comes from renewable sources like photovoltaic systems and wind turbines^5,7,10^. Since the supply of energy coming from renewable sources is not constant, it fluctuates depending on demand and weather conditions, a H_2_ generation system able to operate intermittently has to be adopted. A Polymer electrolyte membrane (PEM) water electrolysis system is a good option to meet the required intermittent operation. These systems are more operational flexible comparing to alkaline and solid oxide electrolysis cell systems^13–15^.

PEM water electrolysis in combination with a CO_2_ hydrogenation reactor is an attractive way for converting electric power coming from renewable sources into CH_4_. Other alternative to generate CH_4_ from renewable energy is the co-electrolysis of H_2_O and CO_2_ in solid oxide cells (SOECs)^16,17^. However, these cells are operated at very high temperatures, which make their design and operation more complicated than PEM electrolysis cells.

The hydrogenation process to convert CO_2_ into CH_4_, also known as the Sabatier reaction, is a highly exothermic process which generates a lot of heat^18–20^. This heat has to be removed from the Sabatier reactor in order to maintain a relatively low temperature. Avoiding high operation temperatures of the Sabatier reactor helps to prevent catalyst sintering which decreases the catalyst lifetime^12,18–21^.

**Figure 1:**
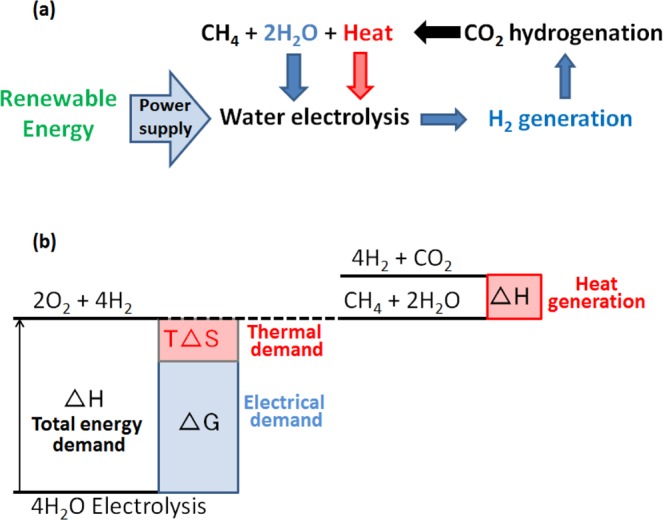
Schematic illustration of the Power-to-Gas concept. **(a)** The system consists of supplying renewable energy to power a water electrolyzer to generate H_2_, then this H_2_ is used to hydrogenate CO_2_ and produce CH_4_ (this process is known as the Sabatier reaction). **(b)** Thermodynamic concept: the theoretical energy required to split liquid H_2_O into gaseous H_2_ and O_2_ is equal to the enthalpy change (Δ*H*_*elec*_) of H_2_O, which is the sum of the Gibbs free energy change (Δ*G*, electrical demand) and entropy change times the temperature (*T*Δ*S*, thermal demand). By exchanging the heat generated by the Sabatier reaction to compensate the water electrolysis thermal demand *T*Δ*S*, the overall efficiency of the system can be improved.

The efficiency of a water electrolyzer–Sabatier reactor system can be improved by thermally coupling the system. To split water into H_2_ and O_2_, the electrolyzer demands electrical and thermal energy. The removed heat from the Sabatier reactor can be supplied to the electrolyzer, which will reduce the thermal energy demand and then the overall efficiency of the system is improved. A schematic illustration of the system concept is shown in Fig. 1a. This PtG system consists of supplying renewable energy to power a water electrolyzer to generate H_2_, and then this H_2_ is used to hydrogenate CO_2_ and produce CH_4_. The efficiency of the system can be increased by exchanging the heat generated during the hydrogenation process of CO_2_ to the water electrolysis process. The thermodynamic concept of the system is shown in Fig. 1b. The theoretical energy required to split liquid H_2_O into gaseous H_2_ and O_2_ is equal to the enthalpy change (Δ*H*_*elec*_) of H_2_O, which is the sum of the Gibbs free energy change (Δ*G*, electrical demand) and entropy change times temperature (*T*Δ*S*, thermal demand). By exchanging the heat generated by the Sabatier reaction to compensate the water electrolysis thermal demand *T*Δ*S*, the overall efficiency of the system can be improved. However, it is necessary to identify the conditions at which a maximum efficiency can be achieved. For this, an exergy analysis of the system is required to identify optimal operating conditions. Exergy valorization analysis is a useful technique to quantify the maximum work potential of a system. This thermodynamic analysis is also used to identify the factors or parameters that affect the efficiency of a process^22–24^.

In this work, an exergy analysis of a water electrolyzer and CO_2_ hydrogenation tandem system is presented. Operating conditions at which high exergy efficiency can be achieved are identified.

## Results and Discussion

### Thermodynamic analysis of CO_2_ hydrogenation process

The equilibrium composition of the CO_2_ hydrogenation process was computed following the Gibbs energy minimization method, see Methods for more details. The main reaction to convert CO_2_ into methane is the Sabatier reaction:

1 $${\rm{Sabatier}}\,{\rm{reaction}}:\,4{{\rm{H}}}_{2}+{{\rm{CO}}}_{2}={{\rm{CH}}}_{4}+2{{\rm{H}}}_{2}{\rm{O}}$$

Also during the CO_2_ hydrogenation process the following side reactions can take place:

2 $${\rm{Methanation}}\,{\rm{reaction}}:\,3{{\rm{H}}}_{2}+{\rm{CO}}={{\rm{CH}}}_{4}+{{\rm{H}}}_{2}{\rm{O}}$$

3 $${\rm{Water}}\,{\rm{gas}}\,{\rm{shift}}\,{\rm{reaction}}:\,{{\rm{H}}}_{2}{\rm{O}}+{\rm{CO}}={{\rm{CO}}}_{2}+{{\rm{H}}}_{2}$$

4 $${\rm{Carbon}}\,{\rm{monoxide}}\,{\rm{reduction}}:\,{\rm{CO}}+{{\rm{H}}}_{2}={\rm{C}}+{{\rm{H}}}_{2}{\rm{O}}$$

5 $${\rm{Carbon}}\,{\rm{dioxide}}\,{\rm{reduction}}:\,{{\rm{CO}}}_{2}+2{{\rm{H}}}_{2}={\rm{C}}+2{{\rm{H}}}_{2}{\rm{O}}$$

**Figure 2:**
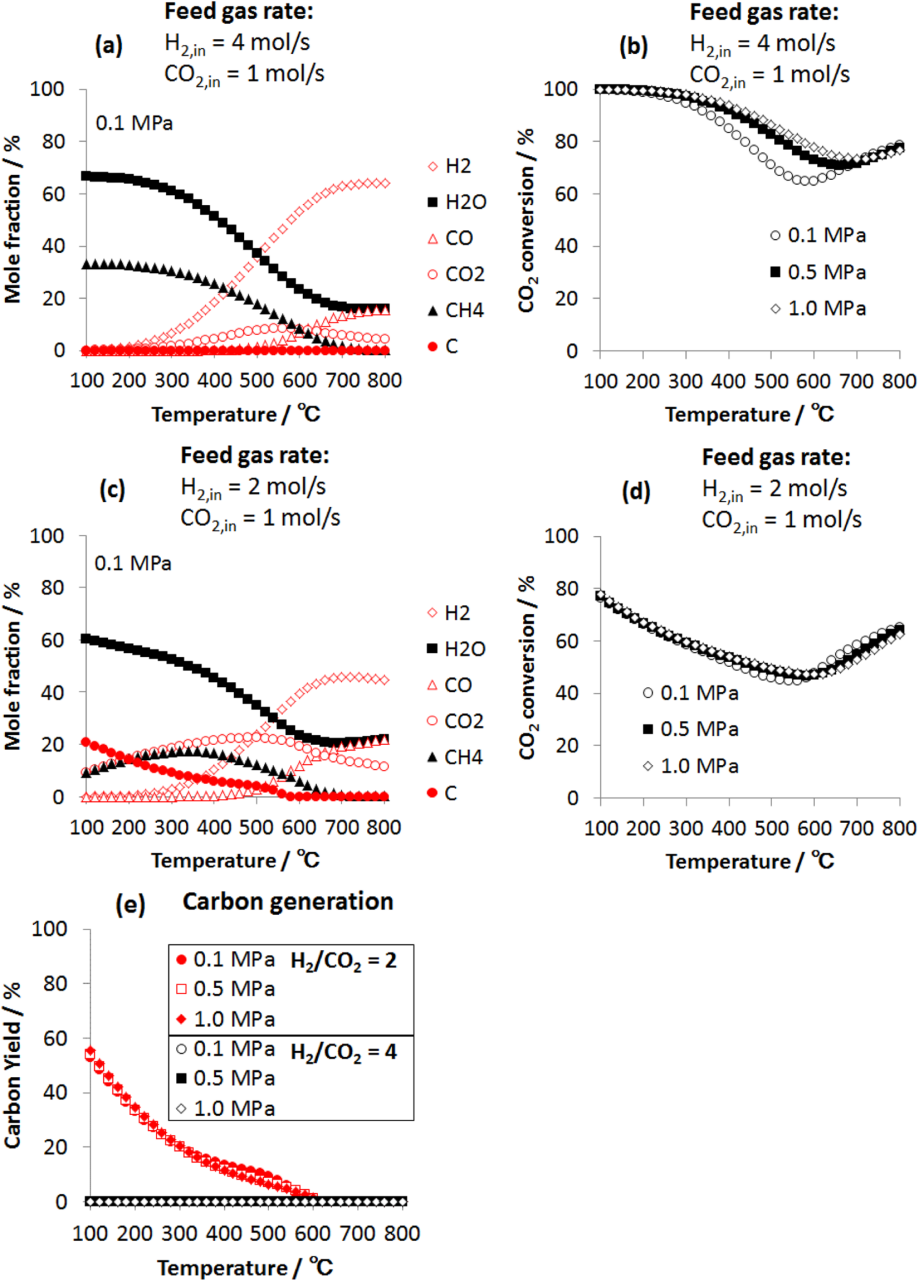
Equilibrium composition of the CO_2_ hydrogenation products as a function of temperature computed following the Gibbs energy minimization method. **(a)** Molar fraction of possible products for a feed gas ratio H_2_/CO_2_ of 4. **(b)** CO_2_ conversion for a feed gas ratio H_2_/CO_2_ of 4. **(c)** Molar fraction of possible products for a feed gas ratio H_2_/CO_2_ of 2. **(d)** CO_2_ conversion for a feed gas ratio H_2_/CO_2_ of 2. **(e)** Effect of the feed gas ratio H_2_/CO_2_ on the carbon generation process.

To analyze the influence of temperature, pressure and feed H_2_/CO_2_ molar ratio on the CO_2_ hydrogenation products, equilibrium compositions were obtained at different conditions. Figure 2a shows the equilibrium composition of the CO_2_ hydrogenation process at H_2_/CO_2_ = 4, 0.1 MPa and different temperatures. It is observed that the largest mole fraction of CH_4_ and H_2_O products are obtained at low temperatures (100–300 °C), indicating that the Sabatier reaction is favored at low temperatures, where a high conversion of CO_2_ into CH_4_ can be achieved. At high temperatures, CH_4_ generation decreases and CO appears which means that the water gas shift reaction is favored. Figure 2b shows the thermodynamic conversion of CO_2_ at 0.1, 0.5 and 1.0 MPa, H_2_/CO_2_ = 4 and different temperatures. A high conversion is observed at low temperatures, it is also noticed that a high conversion can be maintained at higher temperatures by increasing the pressure. The effect of H_2_/CO_2_ molar ratio on the CO_2_ hydrogenation products and CO_2_ conversion is shown in Fig. 2c,d. Under these conditions, the CO_2_ conversion decreases with temperature and the generation of solid carbon (C) is favored. The presence of solid carbon affects the performance of catalyst, since the catalyst can be deactivated when solid carbon is deposited on it. To prevent the deactivation of catalysts, it is important to identify the conditions at which solid carbon can be generated. Figure 2e shows the carbon generation at 4 and 2 H_2_/CO_2_ molar ratios, and at different temperatures and pressures. It is observed that when the H_2_/CO_2_ molar ratio of 4 is not maintained, solid carbon is generated, while at a temperature above 590 °C this carbon generation is not observed under any conditions. This temperature coincides with the operating temperature of the Sabatier reactor used on the International Space Station^11^, probably as a measure to prevent the generation of carbon when the H_2_/CO_2_ ratio of 4 cannot be maintained.

### Exergy analysis of the Sabatier process

**Figure 3:**
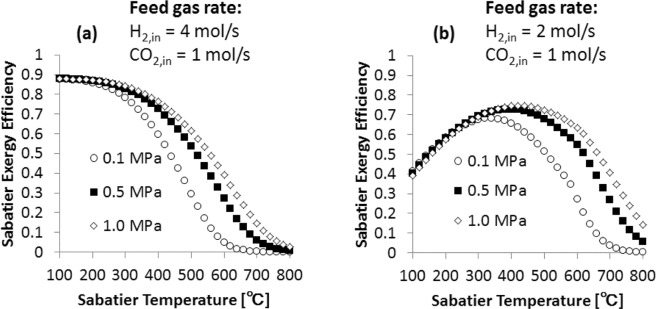
Exergy efficiency of the Sabatier process as a function of temperature for H_2_/CO_2_ of 4 (**a**) and 2 (**b**).

The exergy efficiency of the Sabatier process is defined by

6 $${\eta }_{Sabatier}^{ex}=\frac{C{H}_{4,out}\cdot (E{x}_{C{H}_{4}})}{{H}_{2,in}\cdot (E{x}_{{H}_{2}})}$$

where ${\eta }_{Sabatier}^{ex}$ is the Sabatier exergy efficiency, $E{x}_{C{H}_{4}}$ and $E{x}_{{H}_{2}}$ are the exergy of CH_4_ and H_2_, respectively. Details of the exergy calculations are described in the methods section. Figure 3 shows the exergy efficiency of the Sabatier process for H_2_/CO_2_ molar ratio of 4 (a) and 2 (b) at 0.1, 0.5 and 1.0 MPa as a function of temperature. In the case of the H_2_/CO_2_ molar ratio of 4 (Fig. 3a), the highest efficiency is observed in the temperature range of 100–150 °C and starts decreasing with temperature. In the case of the H_2_/CO_2_ molar ratio of 2 (Fig. 3b), the highest efficiency is observed at 340 °C for 0.1 MPa, while for 0.5 and 1.0 MPa it is observed at 400 °C and 420 °C, respectively. The exergy efficiency of the Sabatier process is proportional to the CH_4_ generation flow rate, and this rate has a high dependency on temperature and H_2_/CO_2_ molar ratio as can be observed in Fig. 2a,c.

### Exergy analysis of the system

**Figure 4:**
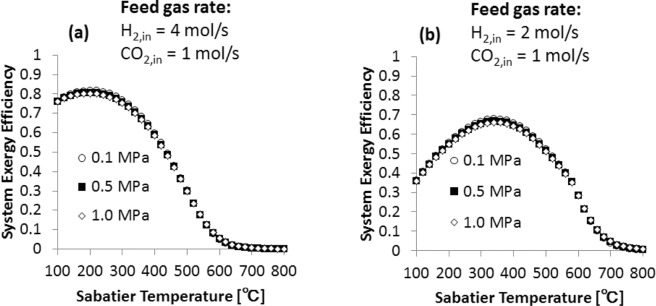
Overall exergy efficiency of the water electrolyzer and CO_2_ hydrogenation tandem system as a function of temperature for H_2_/CO_2_ of 4 (**a**) and 2 (**b**).

The overall exergy efficiency of the system is defined by the following equation:

7 $${\eta }_{overall}^{ex}=\frac{E{x}_{out}}{E{x}_{in}}$$

where ${\eta }_{overall}^{ex}$ is the overall exergy efficiency, $E{x}_{out}$ is the exergy output and $E{x}_{in}$ is the exergy input. Here, $E{x}_{out}$ is equal to $C{H}_{4,out}\cdot (E{x}_{C{H}_{4}})$, while $E{x}_{in}$ corresponds to the electrical energy required to generate hydrogen from water electrolysis, more details on the calculations are given in the methods section. Figure 4 shows the overall exergy efficiency of the system for H_2_/CO_2_ molar ratio of 4 (a) and 2 (b) as a function of Sabatier temperature. In the case of the H_2_/CO_2_ molar ratio of 4 (Fig. 4a), the overall exergy efficiency has a maximum at 200 °C. While in the case of the H_2_/CO_2_ molar ratio of 2 (Fig. 4b), the maximum is found at 340 °C. The maximum overall exergy efficiency is achieved when the thermal exchange between the water electrolyzer and CO_2_ hydrogenation reactor reaches its optimal point. This indicates that the thermal coupling within the system increases the exergy efficiency.

Our exergy valorization analysis identifies the three main parameters that contribute to achieve high exergy efficiencies for the water electrolyzer – CO_2_ hydrogenation system. The first parameter is the H_2_/CO_2_ feed gas molar ratio. When this molar ratio is lower than 4, the methane generation rate decreases and solid carbon is generated, Fig. 2c,e. The second parameter is the Sabatier reaction temperature. CH_4_ generation is favored in the temperature range of 100–300 °C, while at higher temperatures the water gas shift reaction takes, affecting the CH_4_ generation, Fig. 2a. The third parameter that contributes to the enhancement of the system is the thermal coupling of the water electrolyzer and Sabatier reactor. The heat generated by the Sabatier reaction compensates the water electrolysis thermal demand (*T*Δ*S*), which contributes to increase the overall exergy efficiency of the system, Fig. 4a. In the case of co-electrolysis of CO_2_ in H_2_O, it has been shown that this system is also able to achieve high exergy efficiencies to generate H_2_ and CH_4_. However, the water gas shift reaction has a significant effect on the system efficiency^16,17^. As mentioned above, this reaction is favored at high temperatures and this type of CO_2_ co-electrolysis is carried out at high temperatures. In order to mitigate the effect of the water gas shift reaction and increase the efficiency of the system, CH_4_ has to be fed through the cell anode.

Since the focus of the present work is the exergy valorization of the water electrolysis-CO_2_ hydrogenation tandem system, a detailed cost analysis is not included. However, a few remarks can be made on the operating conditions that can have an impact on the system running costs. As mentioned above, maintaining a feed gas rate H_2_/CO_2_ of 4 avoids the generation of solid carbon. The generation of solid carbon affects the catalyst activity and decreases its lifetime, which directly impact the running cost of the system. Another factor that can impact the system running costs is high temperatures. Catalyst sintering is favored at high temperatures and this causes a loss in the catalyst activity^12,18–21^. So that, by operating the system at a relative low temperature (100–300 °C) helps to avoid catalyst sintering.

In summary, our thermodynamic analysis of the water electrolyzer-CO_2_ hydrogenation tandem system exposes the conditions at which high exergy efficiency can be achieved. The results also show that the thermal coupling within the CO_2_ hydrogenation and water electrolysis processes contributes to increase the overall exergy efficiency of the system.

## Methods

In the analysis a steady state and steady flow are assumed. Chemical kinetics effects are not considered in the calculations. All values of enthalpy, Gibbs free energy and entropy for all the species were computed using Aspen Plus V8.8, Aspen Tech^TM^.

### Water electrolysis

The theoretical energy required to split liquid H_2_O into gaseous H_2_ and O_2_ is equal to the enthalpy change (Δ*H*_*elec*_) of H_2_O, which is a function of temperature and pressure and can be calculated with the following equation:

8 $${\rm{\Delta }}{H}_{elec}={\rm{\Delta }}{G}_{elec}+T{\rm{\Delta }}{S}_{elec}$$

where Δ*G* is the Gibbs free energy change and Δ*S* is the entropy change. These thermodynamic parameters are a function of the operative temperature (*T*) and pressure (*P*). The theoretical energy Δ*H* corresponds to the sum of electrical energy demand Δ*G* and thermal energy demand TΔ*S* (*Q*_demand_). Δ*H*, Δ*G* and Δ*S* were calculated using the following equations:

9 $$4{{\rm{H}}}_{2}{\rm{O}}(l)=4{{\rm{H}}}_{2}(g)+2{{\rm{O}}}_{2}(g)$$

10 $${\rm{\Delta }}H=({h}_{{H}_{2}(g)}(T,P)+{h}_{{O}_{2}(g)}(T,P))-({h}_{{H}_{2}O(l)}(T,P))$$

11 $${\rm{\Delta }}G=({g}_{{H}_{2}(g)}(T,P)+{g}_{{O}_{2}(g)}(T,P))-({g}_{{H}_{2}O(l)}(T,P))$$

12 $${\rm{\Delta }}S=({s}_{{H}_{2}(g)}(T,P)+{s}_{{O}_{2}(g)}(T,P))-({s}_{{H}_{2}O(l)}(T,P))$$

Where ${h}_{{H}_{2}(g)}$, ${h}_{{O}_{2}(g)}$ and ${h}_{{H}_{2}O(l)}$ are respectively the enthalpy values of H_2_ and O_2_ in gaseous phase, and H_2_O in liquid phase. ${g}_{{H}_{2}(g)}$, ${g}_{{O}_{2}(g)}$ and ${g}_{{H}_{2}O(l)}$ are respectively the Gibbs free energy of H_2_ and O_2_ in gaseous phase, and H_2_O in liquid phase. ${g}_{{H}_{2}(g)}$, ${g}_{{O}_{2}(g)}$ and ${g}_{{H}_{2}O(l)}$ are respectively the entropy of H_2_ and O_2_ in gaseous phase, and H_2_O in liquid phase. The enthalpy, Gibbs free energy and entropy values were computed using Aspen Plus V8.8, Aspen Tech^TM^.

The exergy input for a water electrolysis process corresponds to the electrical energy demand, which is the minimum work required to split H_2_O into H_2_ and O_2_. Then, the exergy input is:

13 $$E{x}_{in}={\rm{\Delta }}{G}_{elec}={\rm{\Delta }}{H}_{elec}-{Q}_{demand}$$

### CO_2_ hydrogenation process

The equilibrium composition of the reactions was calculated using the Gibbs free energy minimization method, which is a widely used method to perform thermodynamic analysis of reacting systems^20,21,25^. The equilibrium composition and the enthalpy of reaction (Δ*H*_*r*_) for the CO_2_ hydrogenation possible products were computed using Aspen Plus V8.8, Aspen Tech^TM^. The species considered in the analysis are: CH_4_, CO, CO_2_, H_2_O, H_2_ and C (solid carbon). All the species were considered in gas phase, with the exception of C, which was considered in solid phase. The computations were carried out in the temperature range of 100 °C–800 °C and at different pressures.

The thermodynamic conversion of CO_2_, CH_4_ generation and carbon generation were calculated from the equilibrium composition results using the following equations:

14 $$C{O}_{2}\,conversion\,[ \% ]=\frac{C{O}_{2,in}-C{O}_{2,out}}{C{O}_{2,in}}\times (100)$$

15 $$C{H}_{4}\,generation\,[ \% ]=\frac{C{H}_{4,out}}{C{O}_{2,in}}\times (100)$$

16 $$Carbon\,generation\,[ \% ]=\frac{{C}_{out}}{C{O}_{2,in}}\times (100)$$

Where *CO*_*2,in*_ and *CO*_*2,out*_ are the molar flow rate of CO_2_ at inlet and outlet, respectively. *CH*_*4,out*_ is the molar flow rate of CH_4_ at outlet. *C*_*out*_ is the molar flow rate of solid carbon at outlet.

### Exergy calculation

The exergy coming out (*Ex*_*out*_) of the system is calculated based on the CH_4_ generation molar rate:

17 $$E{x}_{out}=C{H}_{4,out}\cdot (E{x}_{C{H}_{4}})$$

The exergy of a specific gas ($E{x}_{i}$) is equal to the sum of physical exergy ($E{x}_{i}^{Ph}$) and chemical exergy ($E{x}_{i}^{Ch}$):

18 $$E{x}_{i}=E{x}_{i}^{Ph}+E{x}_{i}^{Ch}$$

The physical exergy is calculated according the following equation:

19 $$E{x}_{i}^{Ph}={m}_{i}[({h}_{i}-{h}_{0})-{T}_{0}({S}_{i}-{S}_{0})]$$

where *m*_*i*_ is the molar flow rate of the gas, *h*_*i*_ is the enthalpy of the gas at the Sabatier reaction conditions, and *h*_0_ is the enthalpy at the gas at the dead state. T_0_ is the temperature at the dead state. *S*_*i*_ is the entropy of the gas at the Sabatier conditions and *S*_0_ is the gas entropy at the dead state. The dead state conditions are assumed to be 298.15 K and 1 atm. The chemical exergy is calculated using the following equation:

20 $$E{x}_{i}^{Ch}={m}_{i}\cdot (E{x}_{0,i})$$

where $E{x}_{0,i}$ is the standard chemical exergy of the gas.

For the heat flow from the Sabatier reactor (*Q*_*sab*_) to the water electrolysis system, the exergy is calculated as follows:

21 $$E{x}_{heat}={Q}_{demand}(1-\frac{{T}_{elec}}{{T}_{sab}})$$

where ${Q}_{Sab}\ge {Q}_{demand}$, otherwise, there is not heat transfer. *T*_*elec*_ and *T*_*sab*_ are the temperatures of the electrolysis system and Sabatier reactor, respectively.

Calculation of the overall exergy efficiency of the water electrolyzer and CO_2_ hydrogenation tandem system:

22 $${\eta }_{overall}^{ex}=\frac{C{H}_{4,out}\cdot (E{x}_{C{H}_{4}})}{{\rm{\Delta }}{H}_{elec}-{Q}_{demand}(1-\frac{{T}_{elec}}{{T}_{sab}})}$$

## References

[1] The Greenhouse Gases Observing Satellite (GOSAT), Whole-atmosphere monthly CO_2_ concentration, http://www.gosat.nies.go.jp/en/recent-global-co2.html.

[2] Dresselhaus MS, Thomas IL (2001). Alternative energy technologies. Nature.

[3] Chu S, Majumdar A (2012). Opportunities and challenges for a sustainable energy future. Nature.

[4] Turner JA (2004). Sustainable Hydrogen Production. Science.

[5] Crabtree GW, Dresselhaus MS, Buchanan MV (2004). The Hydrogen Economy. Physics Today.

[6] Gahleitner G (2013). Hydrogen from renewable electricity: An international review of power-to-gas pilot plants for stationary applications. Int. J. Hydrog. Energy.

[7] Estermann T, Newborough M, Sterner M (2016). Power-to-gas systems for absorbing excess solar power in electricity distribution networks. Int. J. Hydrog. Energy.

[8] Gotz M (2016). Renewable Power-to-Gas: A technological and economic review. Renewable Energy.

[9] Walker SB, Mukherjee U, Fowler M, Elkamel A (2016). Benchmarking and selection of Power-to-Gas utilizing electrolytic hydrogen as an energy storage alternative. Int. J. Hydrog. Energy.

[10] Simonis B, Newborough (2017). Sizing and operating power-to-gas systems to absorb excess renewable electricity. Int. J. Hydrog. Energy.

[11] Samplatsky, D. J., Grohs, K., Edeen, M., Crusan, J. & Burkey, R. Development and Integration of the Flight Sabatier Assembly on the ISS. *AIAA Paper No*. 2011-5151 (2011).

[12] Shima, A., Sakurai, M., Sone, Y., Ohnishi, M. & Takayuki, A. Development of a CO_2_ Reduction Catalyst for the Sabatier Reaction. *AIAA Paper No*. 2012-3552 (2012).

[13] Carmo M, Fritz DL, Mergel J, Stolten D (2013). A comprehensive review on PEM water electrolysis. *Int. J. Hydrog*. Energy.

[14] Gazey R, Salman SK, Aklil-D’Halluin DD (2006). A field application experience of integrating hydrogen technology with wind power in a remote island location. J. Power Sources.

[15] Irvine JTS (2016). Evolution of the electrochemical interface in high-temperature fuel cells and electrolysers. Nat. Energy.

[16] Ni M (2012). 2D thermal modeling of a solid oxide electrolyzer cell (SOEC) for syngas production by H_2_O/CO_2_ co-electrolysis. Int. J. Hydro. Energy.

[17] Xu H, Chen B, Irvine J, Ni M (2016). Modeling of CH_4_-assited SOEC for H_2_O/CO_2_ co-electrolysis. Int. J. Hydro. Energy.

[18] Brooks KP, Hu J, Zhu H, Kee RJ (2007). Methanation of carbon dioxide by hydrogen reduction using the Sabatier process in microchannel reactors. Chem. Eng. Sci..

[19] Lui Z, Chu B, Zhai X, Jin Y, Cheng Y (2012). Total methanation of syngas to synthetic natural gas over Ni catalyst in a micro-channel reactor. Fuel.

[20] Kiewidt L, Thoming J (2015). Predicting optimal temperature profiles in single-stage fixed-bed reactors for CO_2_-methanation. Chem. Eng. Sci..

[21] Nikoo MK, Amin NAS (2006). Thermodynamic analysis of carbon dioxide reforming of methane in view of solid carbon formation. Fuel Process. Technol.

[22] Taner T (2015). Optimisation processes of energy efficiency for a drying plant: A case of study for Turkey. Energy.

[23] Topal H (2017). Exergy analysis of a circulating fluidized bed power plant co-firing with olive pits: A case study of power plant in Turkey. Energy.

[24] Taner T, Sivrioglu M (2015). Energy-exergy analysis and optimisation of a model sugar factory in Turkey. Energy.

[25] Faungnawakij K, Kikuchi R, Eguchi K (2011). Thermodynamic evaluation of methanol steam reforming for hydrogen production. J. Power Sources.

